# Knowledge, Beliefs, and Behaviors of Turkish Parents about Childhood Vaccination

**DOI:** 10.3390/children10101679

**Published:** 2023-10-12

**Authors:** Zuhal Emlek Sert, Sevcan Topçu, Aysun Çelebioğlu

**Affiliations:** 1 Public Health Nursing Department, Faculty of Nursing, Ege University, Izmir 35040, Turkey; zuhal.emlek.sert@ege.edu.tr; 2 Department of Emergency and Disaster Management, Gordes Vocational School, Manisa Celal Bayar University, Manisa 45750, Turkey; aysun.celebioglu@cbu.edu.tr

**Keywords:** beliefs, childhood vaccination, vaccine hesitancy

## Abstract

Background and Objectives: Vaccination is critical to the prevention and control of infectious disease outbreaks and is also one of the most important public health successes. When it comes to childhood vaccinations, parents’ consent is very important. For this reason, childhood vaccination rates are directly related to the knowledge, beliefs, and behaviors of the parents. Therefore, this study aimed to evaluate the knowledge, beliefs, and behaviors of parents of children aged 0–5 regarding childhood vaccinations and how these beliefs affect their vaccination behaviors. Material and Methods: This descriptive, cross-sectional study was conducted on 302 parents from February to June 2020. Data were collected using a questionnaire form with 26 questions. Sociodemographic characteristics were reported as frequencies, means, and percentages. Multiple regression analysis was utilized to evaluate vaccination behaviors and affective factors. Results: About 87.1% of the parents know that vaccines protect their children from infectious diseases, and 76.8% know that vaccines can have side effects. Although 97.7% of the parents had their children fully vaccinated according to the Extended Immunization Program, 2.3% did not vaccinate their children. Moreover, 98% of the parents trust the information given by healthcare professionals about vaccination. The parents’ beliefs explain 53% (R^2^ = 0.53) of the parents’ child vaccination behavior. Conclusion: This study found that although the knowledge level of parents about vaccines is quite good, negative knowledge and beliefs that may affect vaccination also exist. Considered by parents as a reliable source of information, healthcare professionals should impart their knowledge, beliefs, and concerns regarding immunization.

## 1. Introduction

Vaccines are biological products that protect against diseases by stimulating the immune system [1]. Vaccination is critical to the prevention and control of infectious disease outbreaks and is also one of the most important public health successes [2,3]. To prevent vaccine-preventable infectious diseases from causing epidemics, the number of immunized individuals should remain higher than the population’s social immunity threshold [4].

Despite ongoing efforts toward recovery, a staggering 20.5 million children remained either unvaccinated or under-vaccinated in 2022. Furthermore, the number of children who did not receive any vaccines, often referred to as zero-dose children, reached 14.3 million, which significant increased by 1.4 million compared to 2019 [5]. The global vaccination coverage saw some recovery in 2022. However, approximately 20.5 million children are missing out on lifesaving vaccines globally, i.e., 2.1 million more than that in 2019 [5]. As the world strove to recover from the COVID-19 pandemic, it faced additional challenges in 2022. These challenges include a growing population of children residing in fragile and conflict-affected settings, an alarming rise in misinformation, as well as persistent supply issues [5].

Globally, approximately 20.5 million children do not have access to immunization services [5]; 2% and 3% of children aged 12–23 and 24–35 months, respectively, have no access to vaccination services in Turkey [6]. The World Health Organization (WHO)’s Global Vaccine Action Plan 2011–2020 aims to increase the coverage to over 90% for all vaccines in the national vaccination program in all countries [7]. Despite that, vaccine hesitancy is one of the most prominent problems for vaccination coverage [8,9]. The gradual increase in the number of vaccine refusal cases has led to a decrease in vaccination rates and an increase in the frequency of vaccine-preventable diseases [10,11,12]. The WHO explained in *Ten threats to global health in 2019* that vaccine hesitancy threatens to reverse the progress made in managing vaccine-preventable diseases [8]. The number of families who refused to vaccinate their children was 183 in 2011, which increased to 980 in 2013, 5400 in 2015, 12,000 in 2016, and 23,000 in 2018 [13]. These figures show that the number of parents who have concerns about childhood vaccines has increased in Turkey. If the increase in vaccine refusal continues as rapidly as it has in recent years, the vaccination coverage is estimated to drop to 80% after approximately 5 years. Therefore, a significant increase in the incidence of diseases is very likely, and perhaps eradicated diseases will recur [14].

Regarding childhood vaccinations, obtaining parents’ consent is very important [15]. For this reason, childhood vaccination rates are directly related to the knowledge, beliefs, and behaviors of the parents. The most common reasons for parents to refuse vaccinations include a lack of trust in the content of the vaccine [16], inadequate vaccination information [17,18], fear of vaccine side effects [9,19], incorrect beliefs about vaccinations [11], religious reasons [9,20], not having access to vaccination services [21], cultural differences [22], and/or negative news in the media about vaccinations [23,24]. Recent studies emphasize that the decrease in the vaccination rate is due to parents’ refusal or hesitation on vaccination [10,12,25].

To combat antivaccination beliefs, it is crucial for nurses to conduct scientific research that can enhance societal acceptance and provide viable solutions. Good communication and trust between healthcare providers and parents/individuals seeking vaccination are effective methods to address vaccine hesitancy. Additionally, utilizing mass and social media to inform and educate people about the outcomes of scientific research on vaccines and their effects can accelerate the fight against antivaccine sentiments [10]. Protecting and promoting childhood vaccination rates require an understanding of parents’ values, such as concerns, beliefs, and behaviors toward vaccination. Therefore, this study aimed to evaluate the knowledge, beliefs, and behaviors of parents of children aged 0–5 years as regards childhood vaccinations and how these beliefs affect their vaccination behaviors.

## 2. Materials and Methods

### 2.1. Study Design

A cross-sectional study was conducted from February to June 2020. The required sample size was calculated as 301 parents for a small effect size, α = 0.05, power 0.80, and proportions (one group) using the G power program. Participants who met the following criteria were invited to study: (a) a parent of a child/children aged 0–5 years, (b) able to communicate in the Turkish language, and (c) able to provide informed consent. This study was carried out in the province of Izmir, with 302 parents.

### 2.2. Data Collection

A convenience sampling strategy was used to reach and recruit parents. Data were collected from the Bornova district of İzmir Province through face-to-face survey methods. Bornova district is one of the old residential areas in Izmir and is characterized by an urban population. For data collection, researchers approached the parents in the streets where the population density is relatively high, such as parks, squares, marketplaces, bus stations, metro stations, and shopping centers. Three researchers collected data after explaining the study’s purpose to the potential participants.

To collect the data, a questionnaire form comprising 26 questions was created by the researchers. The prepared questionnaire comprised the following factors: seven questions evaluating sociodemographic characteristics (age, mother’s education status, father’s educational status, mother’s employment status, father’s employment status, income status, and number of children), three questions on the health history of the parents and the child (mothers’ chronic disease status, fathers’ chronic disease status, and children’s chronic disease status), nine questions to determine the extent of the participants’ vaccination knowledge (vaccines protect children from infectious diseases, vaccines provide immunity, children who are not vaccinated can get fatal infectious diseases, vaccines have side effects, rashes and pain in the vaccine area after vaccination, the occurrence of fever in children after vaccination, vaccination can be delayed when the child has a fever, a newborn is given the first vaccine immediately after birth, and the last dose of childhood vaccinations are given in the 8th grade), four questions on the participants’ beliefs about vaccines (vaccines are necessary to protect the child’s health; if my child is not vaccinated, my child can get infectious diseases; babies and children are exposed to too much vaccination; and I trust the information given by healthcare professionals about vaccination), and three questions on behaviors concerning vaccination (the parents’ status of allowing their children to be fully vaccinated according to the Extended Immunization Program, where parents had their children vaccinated, and parents’ status of keeping their children’s vaccination card).

The survey was evaluated for validation as follows: first, the study instrument was sent to 10 experts in the fields of public health and public health nursing for face validity. Expert opinions were used in revising the questionnaire into a simpler version. Second, a pilot study was conducted by 20 parents for their opinions about the survey ambiguity or incomprehensibility.

### 2.3. Data Analyses

Data were analyzed using the Statistical Package for Social Sciences version 20.0. Sociodemographic characteristics were reported as frequencies, means, and percentages. Multiple regression analysis was utilized for the evaluation of vaccination behaviors and affective factors.

### 2.4. Ethical Considerations

To carry out the study, permission was obtained from the institutional review board (approval number: 20-1.1T/23). Families were informed about the study, and their written consent was obtained. Further, written informed consent was obtained from the parents who agreed to participate in the study.

## 3. Results

The study participants consisted of 66.22% females. The mean age of mothers and fathers was 29.9 ± 3.69 and 31.9 ± 4.38, respectively. Of the mothers, 46.4% had children in junior high school level and 80.1% were non-working (Table 1). Of the fathers, 57% had children in junior high school and 100% were employed. More than half (56%) of the parents had an income lower than their expenses, 43.4% had two children, and 7% of the parents and 2.3% of the children had a chronic disease.

Table 2 illustrates the parents’ knowledge of vaccination: 87.1% of the parents know that vaccines protect children from infectious diseases; 53.6% that they provide immunity; 52% know that children who are not vaccinated can acquire fatal infectious diseases; and 76.8% know that vaccines can have side effects. Although 64.9% of the parents know that fever can occur after vaccination, only 30.1% know that rashes and pain may be felt in the vaccine area. Further, 66.6% of the parents think that vaccination can be delayed when the child has a fever. Although 81.5% of the parents know the time of the first childhood vaccination, only 19.5% know the time of the last one.

Table 3 illustrates the behaviors of parents regarding vaccination. Although 97.7% of the parents had their children fully vaccinated according to the Extended Immunization Program (EIP), 2.3% did not vaccinate their children. Almost all parents (99%) had their children vaccinated at the primary healthcare center, and 93.4% of them have kept their children’s vaccination cards.

About 98.7% of the parents believe that vaccines are necessary to protect the child’s health, 95.4% believe that their children will acquire infectious diseases if they are not vaccinated, 5.6% of them believe that babies and children are exposed to too much vaccination (Table 4), and 98% trust the information given by healthcare professionals about vaccination.

The parents’ beliefs that affect the vaccination of their children were evaluated by binary logistic regression analysis (Table 5). The necessity of vaccines for protecting health, whether children who are not vaccinated can acquire infectious diseases, children are exposed to too many vaccines, and trust in healthcare professionals about vaccination were identified as the parents’ beliefs affecting the vaccination of their children. The parents’ beliefs explain 53% (R^2^ = 0.53) of the parents’ child vaccination behaviors.

## 4. Discussion

This study aimed to evaluate the knowledge, beliefs, and behaviors of parents with children aged 0–5 years about childhood vaccinations. About 87.1% of parents know that vaccines protect from infectious diseases, 53.6% know that they provide immunity, and 52% know that children who are not vaccinated can acquire fatal infectious diseases. Several studies reported parents’ views that vaccines protect children from infectious diseases [24,26,27,28]. Although the fact that vaccines protect their children from infectious diseases is known by most of the parents included in the study, almost half of the parents are unaware of the health problems that children may experience if they are not vaccinated. Hence, parents should be informed about the individual health problems that may develop if a vaccine is not administered and the social damage that may develop as well as the benefits of vaccines for children.

However, the parents in this study know that children may experience side effects due to vaccines and that fever may be a side effect, rashes and pain may develop at the vaccination site. Alsalhi et al. (2019) found results consistent with this study; although almost all parents know fever to be a side effect of vaccines, only 16% know about pain, and 13% about redness [18]. Habib et al. (2018) demonstrated that 89% of parents know that pain and fever can occur due to vaccination [26]. However, more than half of parents in this study have been misinformed, believing that vaccination should be delayed when a child has fever. Although most parents know the correct time of the first childhood vaccination, very few of them know the time of the last. A study by Abdullah et al. (2018) revealed that 96.4% of parents knew the time of the first vaccine [20]. Vaccines are administered based on the specified timeline as much as for coverage. Several studies show that encountering side effects such as fever, pain, or swelling at the vaccination site after the first vaccination can negatively affect parents’ subsequent vaccination behaviors [25,29,30,31,32,33,34,35]. Vaccines have been blamed many times throughout history.

Blaming methods not based on scientific basis arise from a lack of understanding on the importance of infectious diseases, vaccine ingredients, and the fact that vaccines can cause diseases. Antivaccine sentiment, which is rapidly spreading in our country and globally, and failure to show sufficient seriousness about infectious diseases may lead to serious epidemics in the near future. They may cause the re-emergence of diseases that have been eradicated but have high morbidity and mortality, such as polio, neonatal tetanus, and diphtheria [34]. For this reason, misinformed parents who may be hesitant or who may reject further vaccination due to side effects should be identified and corrected to maintain immunization.

According to the WHO’s data, vaccination coverage ranges from 95 to 99% in Turkey but is 94% for the second dose of the measles vaccine [36]. Similarly, in this study, 97.7% of parents had fully vaccinated their children according to the EIP. Results from this study show that the minimum coverage rate has been reached for vaccines to achieve social immunity. Despite measles, rubella, and polio eradication studies being conducted in Turkey, 103 measles, 33 rubella, 10 tetanus, and 8 pertussis cases were reported for the first 9 months of 2022 [36]. This situation requires increasing the coverage rates of vaccination and evaluating the factors that cause vaccine rejection or hesitancy. Almost all parents in this study had their children vaccinated at the primary healthcare center and 93.4% kept their children’s vaccination cards. In the results of Özdemir and Kadıoğlu’s research, only 67.4% of parents kept their vaccination cards [28]. The results obtained from this study show that primary healthcare workers are responsible for immunization services in this country, and the prevention of vaccine rejection or hesitancy can only be achieved via primary healthcare workers. Çakırlı et al. revealed the important role of public health nurses, especially those working in primary care, in immunization services. Health professionals should guide families with appropriate education and information campaigns to prevent vaccine hesitancy and rejection [35].

About 98.7% of parents believe that vaccines are necessary to protect health, 95.4% believe that their children will acquire infectious diseases if not vaccinated, and 5.6% believe that babies and children are exposed to too much vaccination. Almost all parents rely on the information given by healthcare professionals about vaccination. These beliefs about vaccination explain 53% (R^2^ = 0.53) of parents’ vaccination behaviors. Previous studies have found that most parents have a strong belief that vaccines are necessary and useful [24,37]. Several studies have shown that parents receiving information from healthcare professionals about vaccination have a positive attitude toward vaccination and that parents who choose vaccination rely on healthcare professionals, such as nurses and doctors. Pardede et al. (2020) found that nurses’ attitudes positively affect mothers’ motivations regarding vaccination [38]. This study demonstrates the beliefs that vaccination is necessary and that children will get infected with infectious diseases if vaccination is not applied. Moreover, relying on the information provided by healthcare workers positively affects the behaviors of parents regarding the vaccination of their children and demonstrates that the belief that children are exposed to too much vaccination negatively affects vaccination. In this study, the reason for the high immunization rates of children suggests that most parents are well educated and have a good income. As a result of this study, vaccine rejection was found to be higher in families with higher income and educational levels. In a study among pediatricians in the United States, >30% of pediatricians refused immunization. Physicians serving wealthier and better-educated families have been reported to experience more vaccine rejections [39]. A study of mothers with high socioeconomic status revealed the increasing rates of vaccine rejection in this group of parents [40]. Some studies show that a low educational level also increases vaccine rejection. However, child immunization rates may be adversely affected because of antivaccination beliefs during the COVID-19 pandemic process, and future research should be carried out on this issue.

Public health nurses, who work in primary healthcare institutions and practice vaccinations, should evaluate beliefs affecting parents’ vaccination behaviors. Primary healthcare workers, especially public health nurses, should be included in campaigns and training to increase vaccination rates [41].

The present study demonstrates that parents believe that vaccination is necessary and that children will get infected with infectious diseases if vaccination is not applied. Moreover, relying on the information provided by healthcare workers positively affects the behaviors of parents regarding the vaccination of their children and demonstrates that the belief that children are exposed to too much vaccination negatively affects vaccination. Public health nurses are the occupational group that can best observe the reasons for antivaccination in society, as they communicate with families one-to-one and interact with patients the most. Public health nurses, who work in primary healthcare institutions and practice vaccination, should evaluate the beliefs affecting parents’ vaccination behaviors. This is because taking action depends on an individual’s perception of the benefits and obstacles associated with his or her own health behavior. According to the Health Belief Model in health institutions, health personnel and parents should enhance their sensitivity to perceptions of vaccination and recognize their obstacles in improving health; taking continuous initiatives toward this end will then increase the success [42]. It is recommended that future studies on this subject be conducted to understand parents’ opposition to vaccination. Primary healthcare workers, especially public health nurses, should be included in campaigns and training to increase vaccination rates.

The study findings should be interpreted in the light of several limitations. First, this study was conducted with urban parents in İzmir City. Thus, the findings do not represent rural parents. Moreover, most parents were women in this study. The findings may not be representative of all Turkish parents: this was a cross-sectional study, and data were collected via random sampling from a densely populated street. This study evaluated the knowledge level, beliefs, and behaviors of parents with 0–5-year-old children about vaccines. However, whether other children had any underlying disease or had vaccine side effects was not questioned. Although the age range of children is not very wide, the retrospective recall factor and the expected responses of parents with a 5-year-old child should also be carefully considered. This may have led to inconsistencies among parents, particularly in recalling experiences with the vaccine. In addition, a disproportionate distribution was observed between the male and female parent groups. A larger study population, more homogeneous groups, and multicenter studies that represent the whole country can be conducted. Furthermore, the data collection process coincided with the beginning of the pandemic and the period when the curfew was being implemented. Therefore, the sample size could be limited. As a result, the findings cannot be generalized to the whole Turkish population and should be cautiously interpreted. Additionally, this study has a large confidence interval observed in multiple regression analysis. In a larger study, this CI is expected to be smaller.

## 5. Conclusions

Immunization is one of the main preventive health strategies for preventing childhood morbidity and mortality worldwide. Thus, the parents’ beliefs that directly affect their vaccination behaviors should be evaluated. This study found that although the knowledge level of parents about vaccines is quite good, negative knowledge and beliefs that may affect vaccination do not exist in Turkey. For this reason, public health nurses who directly interact with parents in primary healthcare institutions need to evaluate parents’ knowledge and beliefs about vaccination. Considering them as a reliable source of information, healthcare professionals should consider their knowledge, beliefs, and concerns regarding immunization. Moreover, increasing parents’ knowledge of and positive beliefs about vaccination will increase the immunization rates. This is the only method to prevent vaccination refusal, hesitancy, and delays.

Furthermore, this study demonstrates that as trust in healthcare workers increases, the compliance of parents and consequently their participation in immunization services increase. Public health nurses are knowledgeable and can employ effective communication techniques for the development of trust in healthcare workers. Therefore, public health nurses should be supported via up-to-date in-service training on both vaccination and effective communication techniques. Qualitative studies are recommended to determine factors affecting parents’ trust in healthcare workers regarding vaccination.

As a public service, the public should be informed about diseases that can be prevented by vaccination, and legal regulations should be made on this subject. Public health nurses working in primary healthcare institutions should take responsibility for educating parents and implementing policies that will eliminate vaccine hesitancy. Information pollution about vaccination in the mass media should be combated, and public awareness should be raised in light of scientific facts regarding vaccination.

## Figures and Tables

**Table 1:** Parents’ sociodemographic characteristics (N = 302).

Sociodemographic Characteristics	N (%)	(%)
Mothers’ educational status		
Illiterate	4 (1.3)	1.3
Literate	24 (7.9)	7.9
Elementary school	118 (39.1)	39.1
Junior high school	140 (46.4)	46.4
University and above	16 (5.3)	5.3
Fathers’ educational status		
Illiterate	1 (0.3)	0.3
Literate	10 (3.3)	3.3
Elementary school	96 (31.8)	31.8
Junior high school	172 (57.0)	57
University and above	23 (7.6)	7.6
Mothers’ employment status		
Working	60 (19.9)	19.9
Non-working	242 (80.1)	80.1
Fathers’ employment status		
Working	302 (100.0)	100.0
Non-working	0 (0.0)	0.00
Income status		
Income more than expenses	4 (1.3)	1.3
Income less than expenses	169 (56.0)	56.0
Income equal to expenses	129 (42.7)	42.7
Number of children 1 2 3 4>	96 (31.8) 131 (43.4) 52 (17.2) 23 (7.6)	31.8 43.4 17.2 7.6
Mothers’ chronic disease status		
Yes	23 (7.6)	7.6
No	279 (92.4)	92.4
Fathers’ chronic disease status		
Yes	21 (7.0)	7
No	281 (93.0)	93
Children’s chronic disease status		
Yes	7 (2.3)	2.3
No	295 (97.7)	97.7

**Table 2:** Parents’ knowledge about vaccination (N = 302).

Questions	Yes	No
	N (%)	N (%)
Vaccines protect children from infectious diseases	263 (87.1)	39 (12.9)
Vaccines provide immunity	162 (53.6)	140 (46.4)
Children who are not vaccinated can acquire fatal infectious diseases	157 (52.0)	145 (48.0)
Vaccines have side effects	232 (76.8)	70 (23.2)
Rashes and pain in the vaccination area after vaccination	91 (30.1)	211 (69.9)
Fever can occur in children after vaccination	196 (64.9)	106 (35.1)
Vaccination can be delayed when the child has a fever	201 (66.6)	101 (33.4)
A newborn is given the first vaccine immediately after birth	246 (81.5)	56 (18.5)
The last dose of childhood vaccinations is given in the 8th grade	59 (19.5)	243 (80.5)

**Table 3:** Behaviors of parents regarding vaccination (N = 302).

Parents’ Behaviors	N (%)
The parents’ status of having their children fully vaccinated according to the Extended Immunization Program	
Yes	295 (97.7)
No	7 (2.3)
Where parents had their children vaccinated	
Primary Healthcare Center	299 (99.0)
Hospital	3 (1.0)
Parents’ status of keeping their children’s vaccination card Yes No	282 (93.4) 20 (6.6)

**Table 4:** Beliefs of parents about vaccines (N = 302).

Parents’ Beliefs	N (%)
Vaccines are necessary to protect child health Agree Not agree	298 (98.7) 4 (1.3)
If I had not my child vaccinated, my child can acquire infectious diseases Agree Not agree	288 (95.4) 14 (4.6)
Babies and children are exposed to too much vaccination Agree Not agree	17 (5.6) 285 (94.4)
I trust the information given by healthcare professionals about vaccination Agree Not agree	296 (98.0) 6 (2.0)

**Table 5:** Multiple regression analysis of parents’ beliefs on vaccination behaviors (N = 302).

Variables	The Parents’ Status of Fully Vaccinating Their Children			
	*p*	Exp (β)	%95 CI	
Vaccines are necessary to protect child health	**0.043**	13.77	1.09	173.97
If my child is not vaccinated, my child can get infectious diseases	**0.044**	7.5	1.07	55.38
Babies and children are exposed to too much vaccination	**0.009**	0.06	0.01	0.51
I trust the information given by healthcare professionals about vaccination.	**0.021**	20.36	1.57	263.35
−2 Log likelihood	33.26			
Cox–Snell R Square	0.10			
Nagelkerke R Square	0.53			
X^2^	33.27			
*p*	**0.001**			

## Data Availability

The original contributions presented in the study are included in the article. Further inquiries can be directed to the corresponding authors.
